# Ammonia-Responsive Luminescence of Ln^3+^-β-diketonate Complex Encapsulated within Zeolite Y

**DOI:** 10.3390/molecules24040685

**Published:** 2019-02-14

**Authors:** Yuchen Deng, Peng Li, Yige Wang, Tianren Wang, Huanrong Li

**Affiliations:** Hebei provincial Key Lab of Green Chemical Technology and High Efficient Energy Saving, School of Chemical Engineering and Technology, Hebei University of Technology, GuangRong Dao 8, Hongqiao District, Tianjin 300130, China; dengyuchen816@163.com (Y.D.); lipeng@hebut.edu.cn (P.L.); 15122485497m@sina.cn (T.W.)

**Keywords:** Ln^3+^-β-diketonate complex, zeolite Y, luminescence, ammonia-responsive

## Abstract

Assembling Ln^3+^(HPBA_n_) (Ln = Eu or Tb, HPBA = N-(2-pyridinyl)benzoylacetamide) in the cavities of zeolite Y (ZY) via the “ship-in-a-bottle” strategy leads to the formation of novel luminescent composite, Ln(HPBA_n_)@ZY, whose luminescence can be easily modulated by ammonia on the basis of the energy level variation of HPBA after deprotonation process. Additionally the bimetallic complex doping sample, Eu_0.5_Tb_0.5_(HPBA_n_)@ZY, show great potential as self-referencing luminescent sensor for detecting low ammonia concentration of 10^−12^–0.25 wt%.

## 1. Introduction

Stimuli-responsive luminescent materials have attracted intensive interest in a wide range of research fields, including sensors, probes, smart optical devices, to name a few [1,2,3,4,5,6]. Lanthanide-based stimuli-responsive materials are highly interesting and desirable due to their unique optical properties such as narrow emission bands and long decay time of the excited state [7,8,9,10,11,12]. This kind of materials possess the ability of changing their structure and hence their luminescent properties when stimulated by external factors such as pH [13,14,15], light [16,17,18], temperature [19,20] and chemicals [10]. However, most of the stimuli-responsive systems rely heavily on the design and synthesis of complicated molecules and assemblies, which normally requires multi-step syntheses and tedious purification procedures. Therefore, it is important and desirable to develop a facile, cost-effective and environmentally friendly method for the construction of stimuli-responsive luminescent materials. Recently, we have developed a facile strategy to prepare stimuli-responsive luminescent materials by simply incorporating lanthanide compounds into hydrogels [21] or inorganic matrices such as zeolites [22,23] and layered clays [24], the luminescence of which can be readily modulated by ammonia and other chemicals [25,26]. For instance, the on/off luminescence of europium (III) complexes encapsulated within the channels of nanosized zeolite L controlled by base/acid have been recently reported by our group [22], based on the observation that the high proton strength inside the channels of Eu^3+^-doped zeolite L can be detrimental to the luminescence of the hosted europium complexes and alkaline molecules such as triethylamine can neutralize the acidic sites and therefore can switch on the luminescence. This strategy to obtain stimuli-responsive luminescent systems is simple, cost-and time-effective, easy to be scaled up, and environmentally friendly because it does not require intensive organic synthesis procedure.

Herein, we developed the ammonia-responsive luminescent systems by encapsulating Ln(HPBA_n_) (Ln = Eu or Tb, HPBA = N-(2-pyridinyl)benzoylacetamide) within the cavities of ZY. Fascinatingly, the emission color of Eu_0.5_Tb_0.5_(HPBA)_n_@ZY varies from green to red after treatment with NH_3_ vapor, since the introduction of NH_3_ into Eu_0.5_Tb_0.5_(HPBA_n_)@ZY can change the chemical properties of the ligand, thus the triplet energy level of the ligand matches well with the ^5^D_0_ energy level of Eu^3+^. Eu_0.5_Tb_0.5_(HPBA)_n_@ZY shows promise in the ammonia detection application. Ammonia is commonly used in various industries such as agriculture, medicine and food industries, etc. [27,28]. However, ammonia can causes serious harm to the human body [29,30], and dissolved ammonia at concentrations above 25 μg/L is also particularly harmful to organisms [27]. Therefore, ammonia detection is of great importance in the environmental monitoring field [31]. The traditional methods for ammonia detection, including electrochemical sensing, HPLC and mass spectrometry coupled with gas chromatography, often require complicated and time-consuming pretreatments and specialized instruments, which limit their widespread application [32,33,34,35,36].

## 2. Results and Discussion

**Figure 1:**
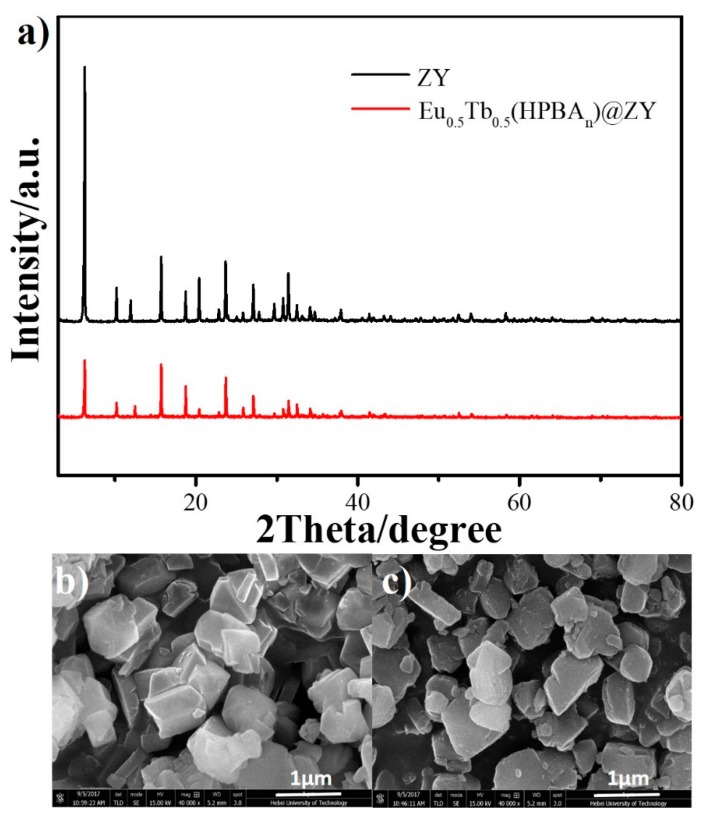
XRD patterns (**a**) and SEM images of ZY (**b**) and Eu_0.5_Tb_0.5_(HPBA_n_)@ZY (**c**).

The hybrid composites Eu(HPBA_n_)@ZY, Tb(HPBA_n_)@ZY and Eu_0.5_Tb_0.5_(HPBA_n_)@ZY were prepared by using the so-called “ship-in-the-bottle” method [37,38]. Firstly Ln^3+^-exchanged ZY (Ln^3+^@ZY) was prepared through an ion-exchange process, and then Ln(HPBA_n_)@ZY was obtained through the formation of Ln(HPBA_n_) complex within the channels of Ln^3+^@ZY. The Ln^3+^ loading in Eu_0.5_Tb_0.5_(HPBA_n_)@ZY was roughly analyzed to be 8.5 wt% through the EDTA titration method, while the HPBA content in the composite was approximately 3.5 wt% as determined by elemental analysis. In addition, no obvious changes in the XRD patterns and morphology of ZY (Figure 1) can be observed after encapsulation with Ln^3+^ complexes, indicating that the reaction is not detrimental to the ZY framework.

**Scheme 1:**
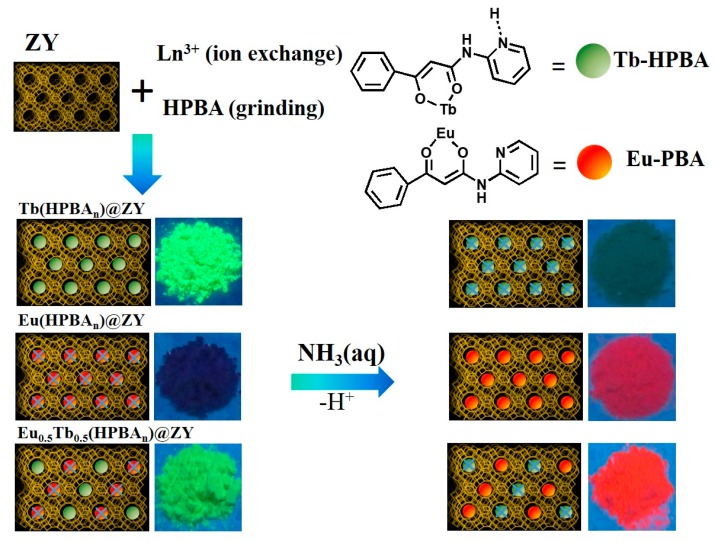
Illustration for the preparation of the hybrid composite Tb(HPBA_n_)@ZY, Eu(HPBA_n_)@ZY Eu_0.5_Tb_0.5_(HPBA_n_)@ZY, as well as the influence of NH_3_ (aq) on the emission color of the hybrid composite.

As mentioned in the previous report [2,39,40], the HPBA ligand in the Ln^3+^ complexes exists as an enol tautomer, which possess the ability to sensitize Tb^3+^, while the deprotonated form (named PBA) can efficiently transfer energy to Eu^3+^ (see Scheme 1). In the acidic environment of zeolite Y [22,41], both Tb(HPBA_n_)@ZY and Eu_0.5_Tb_0.5_(HPBA_n_)@ZY show bright green emission light under UV-light illumination as shown in Scheme 1 although almost equal amounts of Eu^3+^ and Tb^3+^ in Eu_0.5_Tb_0.5_(HPBA_n_)@ZY can be confirmed by the EDX spectrum shown in Figure S1, whereas sample Eu(HPBA_n_)@ZY does not show any light under UV-light. Various changes in the luminescence of all the samples(Eu(HPBA_n_)@ZY, Tb(HPBA_n_)@ZY and Eu_0.5_Tb_0.5_(HPBA_n_)@ZY) upon treatment with ammonia vapors as displayed in Scheme 1, where the bright green luminescence for Tb(HPBA_n_)@ZY almost disappeared and obvious red luminescence for Eu(HPBA_n_)@ZY appeared, whereas the bright green luminescence changed to bright red luminescence for the co-doped sample Eu_0.5_Tb_0.5_(HPBA_n_)@ZY. Such emission color variation under ammonia treatment is consistent with that of the previously reported pure Ln^3+^-HPBA (Ln = Eu or Tb) complexes [2,39,40].

**Figure 2:**
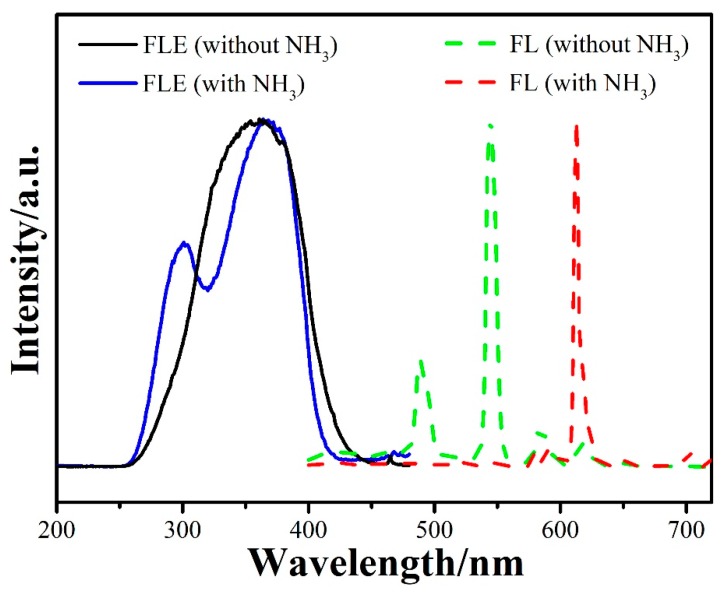
FL excitation (solid line) and FL emission spectra (dot line) of Eu_0.5_Tb_0.5_(HPBA_n_)@ZY before and after treatment with NH_3_ vapor.

In order to search for the explanation for the alteration in the luminescence of all the samples treated with ammonia vapors. Excitation and emission spectra were measured and shown in Figure S2 and Figure 2. A broad band ranging from 250 to 450 nm in the excitation of Tb(HPBA_n_)@ZY was observed by monitoring the ^5^D_4_→^7^F_5_ transition at 540 nm, attributed to the absorption of the ligand, excitation into the ligand absorption at 369 nm led to the characteristic emission of Tb^3+^ ions, implying effective energy transfer from ligand to Tb^3+^ ions. A similar absorption band but with less-resolved structure appeared in the excitation spectrum of Eu(HPBA_n_)@ZY, and the strong absorption band attributed to the Eu^3+^ itself as also observed, five weak sharp lines characteristic of Eu^3+^ ions was observed in its corresponding emission spectrum, the presence of strong Eu^3+^ absorption and the weak emission lines of Eu^3+^ ions imply the much less energy transfer from ligand to Eu^3+^, which can explain why no obvious red luminescence can be observed when Eu(HPBA_n_)@ZY was illuminated by a UV-light. Both the excitation and emission spectra of Eu_0.5_Tb_0.5_(HPBA_n_)@ZY are the same as those of Tb(HPBA_n_)@ZY before ammonia treatment, and no emission lines characteristic of Eu^3+^ ions can be observed despite the presence of Eu^3+^ ions in the sample, which is in good agreement with the observed bright green luminescence under UV-light shown in Scheme 1. Once contacting the sample with ammonia vapors, strong emission lines characteristic of Eu^3+^ ions together with relatively weak emission lines from Tb^3+^ were observed for Eu_0.5_Tb_0.5_(HPBA_n_)@ZY, explaining why the emission color of Eu_0.5_Tb_0.5_(HPBA_n_)@ZY changes from green to red when treated with ammonia.

To explain the ammonia-responsive luminescence of the sample, we have to carefully consider the nature of the ligand HPBA and the properties of the microenvironment inside the cavities of ZY. As well established in the previously reported studies, HPBA can be an effective sensitizer for Tb^3+^ ions under neutral or slight acid conditions and for Eu^3+^ ions under alkaline conditions due to the modulated energy level of the ligand by acid/base vapor [39]. For the Ln^3+^-doped zeolites, abundant acidic sites can be available inside the channels and cavities [22,41], which can be responsible for the bright green luminescence of Tb(HPBA_n_)@ZY and the very weak luminescence of Eu(HPBA_n_)@ZY as well as the distinct changes in the luminescence behaviors upon treatment with ammonia vapors. Such emission color variation can be ascribed to the reaction between the ligand HPBA and ammonia, which leads to the deprotonation of HPBA, and the as-formed ligand PBA is more suitable for sensitizing Eu^3+^ rather than Tb^3+^ (see Scheme 1) [2,39,40].

**Figure 3:**
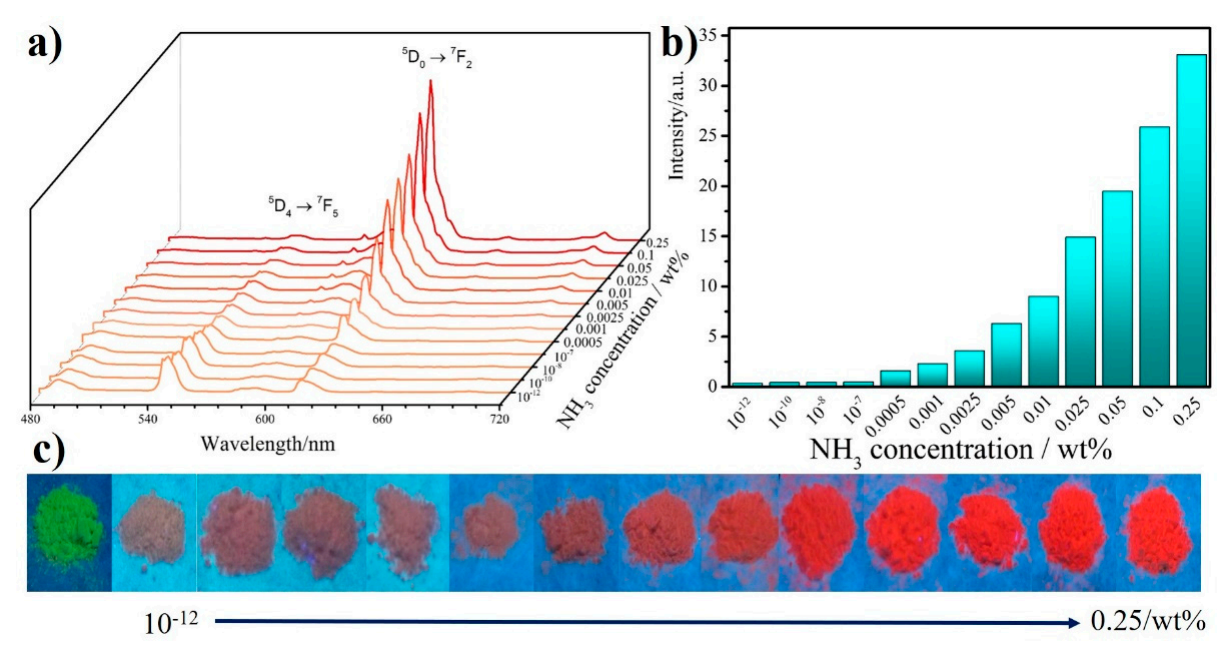
The emission spectra (**a**, λ_ex_=365 nm) and emission intensity ratio of ^5^D_0_→^7^F_2_ transition of Eu^3+^ to ^5^D_4_→^7^F_5_ transition of Tb^3+^ (I_Eu_/I_Tb_, **b**) of Eu_0.5_Tb_0.5_(HPBA_n_)@ZY after treatment with various concentration of aqueous ammonia, as well as the corresponding digital photograph of Eu_0.5_Tb_0.5_(HPBA_n_)@ZY (**c**).

**Figure 4:**
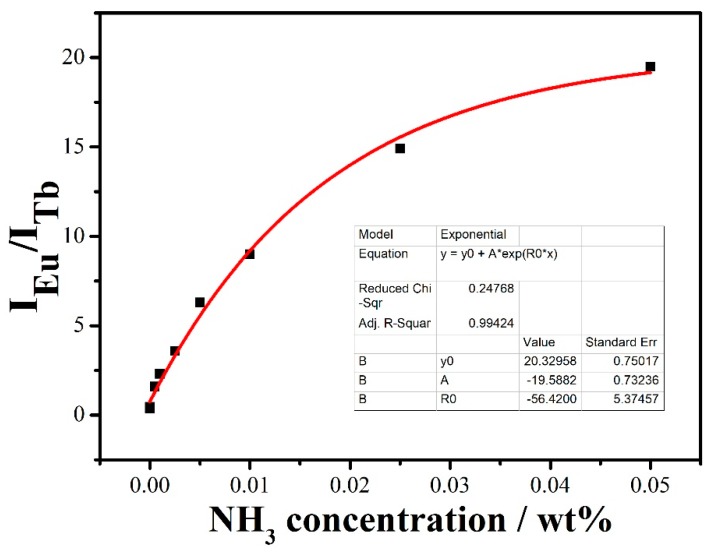
The variation curve of I_Eu_/I_Tb_ versus the concentration of aqueous ammonia (c_a_).

We further investigated the luminescence of Eu_0.5_Tb_0.5_(HPBA_n_)@ZY when it was exposed to the vapors of various concentration of aqueous ammonia. The luminescence performance of Eu_0.5_Tb_0.5_(HPBA_n_)@ZY as a function of the concentration of aqueous ammonia (c_a_) was investigated, as shown in Figure 3 and Figure S3 (Supporting Information). We surprisingly found that even a very low c_a_ (10^−12^ wt%) can turn the emission color of Eu_0.5_Tb_0.5_(HPBA_n_)@ZY to red (see Figure 3 and Figure S3). Furthermore, according to the emission spectra of Eu_0.5_Tb_0.5_(HPBA_n_)@ZY shown in Figure 3a, we found that the ^5^D_0_→^7^F_2_ transition intensity of Eu^3+^ (I_Eu_) increases gradually while the ^5^D_4_→^7^F_5_ transition of Tb^3+^ (I_Tb_) decreases gradually with the increase of c_a_. As a result, the emission intensity ratio I_Eu_/I_Tb_ increases gradually, as shown in Figure 3b. Besides, the emission color variation of Eu_0.5_Tb_0.5_(HPBA_n_)@ZY towards different c_a_ (10^−12^–0.25 wt%) can also be easily observed by naked eyes under 365 nm UV lamp illumination (see Figure 3c and Figure S3). The quantitative relationship between I_Eu_/I_Tb_ and c_a_ was also established, as shown in Figure 4. The curve can be exponentially fitted in the c_a_ range of 10^−12^ to 0.25 wt% (R^2^ = 0.994), and the fitted equation was shown as follows:

I_Eu_/I_Tb_ = 20.33 − 19.59 × exp(−56.42c_a_) (1)

In total, such dual-emission luminescent sensor is fast-response, sensitive and self-calibrating. Actually, we have also investigated the emission color response of Eu_0.5_Tb_0.5_(HPBA_n_)@ZY with several other organic amines (including triethylamine (Et_3_N), ethanediamine (En), aniline, benzylamine, *tert*-butylamine (t-BuNH_2_), *n*-butylamine (n-BuNH_2_) and N-methylaniline, see Figure S4, Supporting Information). Obviously, not all the organic amines would give the same effect. In particular, ammonia gives more considerable changes for the luminescence of Eu_0.5_Tb_0.5_(HPBA_n_)@ZY. So this luminescent sensor displays excellent selectivity for ammonia.

## 3. Materials and Methods

### 3.1. General Information

2-Aminopyridine (99%), ethyl benzoylacetate (95%), EuCl_3_·6H_2_O (99.9%) and TbCl_3_·6H_2_O (99.9%) were purchased from Beijing HWRK Chemical Co. Ltd. (Beijing, China). Zeolite Y (ZY) were obtained from Sigma-Aldrich (Saint Louis, MO, USA). All reagents and solvents were used without further purification. Fourier transform infrared spectroscopy (FT-IR) spectra were recorded in the spectral range from 4000 to 400 cm^−1^ with a Vector 22 spectrophotometer (Bruker, Karlsruhe, Germany) by using pressed KBr pellets for solid samples. ^1^H-NMR spectra were recorded at room temperature, using perdeuterated solvents as internal standards, on a NMR system VX300 (Varian Mercury, Palo Alto, CA, USA). Elemental analysis was performed on an Elementar Vario EI system (Thermo Electron Corporation, Waltham, MA, USA). SEM images were obtained from an S-4300 FE-SEM (Hitachi, Tokyo, Japan) at an acceleration voltage of 10 kV. X-Ray diffraction data were recorded on a Bruker D8 diffractometer with Cu-Ka radiation. (Bruker, Karlsruhe, Germany) The steady-state luminescence spectra and the life-time measurements were measured on an FS920P near-infrared spectrometer (Edinburgh Instruments, Edinburgh, England), with a 450 W xenon lamp as the steady-state excitation source, a double excitation monochromator (1800 lines mm^−1^), an emission monochromator (600 lines mm^−1^), and a semiconductor cooled RMP928 photomultiplier tube (Hamamatsu, Hamamatsu, Japan).

### 3.2. Synthesis of the β-diketone Ligand HPBA

N-(2-Pyridinyl)benzoylacetamide (HPBA) was synthesized according to the literature procedure [2]. Ethyl benzoylacetate was dissolved in *p*-xylene and 2-aminopyridine was added, the mixture was refluxed at 135 °C for 10 h under vigorous stirring. The product was precipitate from the reaction mixture after adding petroleum ether. The crude product was purified by recrystallization in ethanol, and HPBA was obtained as a white powder in 65% yield. FT-IR (KBr): 3030 cm^−1^ (ν_C-H_), 1442 cm^−1^ (ν_C = N_), 1192 cm^−1^ (ν_C-N_), 730–770 cm^−1^ (ν_C-H_) (see Figure S5, Supporting Information). ^1^H-NMR (400 MHz, CDCl_3_): δ = 4.16 (s, 2H), 7.07 (t, 1H), 7.53 (t, 2H), 7.65 (t, 1H), 7.72 (t, 1H), 8.06 (d, 2H), 8.20 (d, 1H), 8.34 (d, 1H), 9.53 (s, 1H).

### 3.3. Preparation of the Ln^3+^-Exchanged ZY

ZY (1 g) was dispersed in an ethanol solution of LnCl_3_ (0.1M) (Ln = Eu or Tb), and then the mixture was stirred at 60 °C for 24 h. The product was recovered by centrifugation, washed with deionized water for three times and dried at 70 °C, which was donated as Ln^3+^@ZY.

### 3.4. Preparation of the Luminescent Hybrid Composite Ln^3+^(HPBA_n_)@ZY

Ln^3+^@ZY (200 mg) and HPBA (100 mg) were manually ground for 30 min, and the product was washed with dichloromethane three times following by drying at 70 °C for 12 h.

### 3.5. Exposure to Aqueous Ammonia (Concentration from 10^−12^ to 0.25 wt%)

The powder samples of Ln^3+^(HPBA_n_)@ZY were used to detect ammonia concentration. For each experiment, the composite (200 mg) was put in a small bottle and exposed to aqueous ammonia. The powder samples and the small bottle were placed into a sealed container (about 100 mL), which contained about 5 mL liquid for 1 h.

## 4. Conclusions

In summary, we have prepared novel luminescent composites by doping lanthanide complexes to the cavities of ZY through the so-called “ship-in-a-bottle” method. Samples of both Tb(HPBA_n_)@ZY and Eu_0.5_Tb_0.5_(HPBA_n_) @ZY show green emission under UV-light illumination, while no obvious emission can be observed for Eu(HPBA_n_)@ZY. The presence of ammonia vapors can make Eu_0.5_Tb_0.5_(HPBA_n_)@ZY and Eu(HPBA_n_)@ZY emit red emission while severely quenching the luminescence of Tb(HPBA_n_)@ZY. This is because the ammonia can alter the energy level of the ligand through deprotonation. In addition, Eu_0.5_Tb_0.5_(HPBA_n_)@ZY exhibits high sensitivity to low-concentrations of ammonia in aqueous solution, with exponential relationships between the emission intensity ratio I_Eu/Tb_ and the concentration of ammonia in the range of 10^−12^–0.5 wt%, showing advantages like simple preparation, fast response and high sensitivity.

## Supplementary Materials

The following are available online. Figure S1: The EDX spectrum of Eu_0.5_Tb_0.5_(HPBA_n_)@ZY; Figure S2: Excitation (green solid line) and emission spectra (green dot line) of Tb(HPBA_n_)@ZY before treatment with NH_3_ vapor; excitation (black solid line) and emission spectra (black dot line) of Tb(HPBA_n_)@ZY after treatment with NH_3_ vapor (a). Excitation (blue solid line) and emission spectra (blue dot line) of Eu(HPBA_n_)@ZY before treatment with NH_3_ vapor; excitation (red solid line) and emission spectra (red dot line) of Eu(HPBA_n_)@ZY after treatment with NH_3_ vapor (b); Figure S3: The corresponding CIE 1931 coordinates of Eu_0.5_Tb_0.5_(HPBA_n_)@ZY after treatment with different concentration of ammonium hydroxide; Figure S4: Digital photographs of Eu_0.5_Tb_0.5_(HPBA_n_)@ZY upon contact with amines for 10 min under near UV irradiation at 365 nm; Figure S5: FTIR spectrum of HPBA.
