# Supplementary material for: Ammonia-Responsive Luminescence of Ln3+-β-diketonate Complex Encapsulated within Zeolite Y
Source: Molecules. 2019 Feb 14;24(4):685. doi: 10.3390/molecules24040685 (PMC6412632; doi:10.3390/molecules24040685)
Supplement: Supplementary file 1 [file molecules-24-00685-s001.pdf]

Supporting Information

Ammonia-Responsive Luminescence of Ln^{3+}-β-diketonate Complex Encapsulated within Zeolite Y

Yuchen Deng, Peng Li, Yige Wang *, Tianren Wang and Huanrong Li *

Hebei provincial Key Lab of Green Chemical Technology and High Efficient Energy Saving, School of Chemical Engineering and Technology, Hebei University of Technology, GuangRong Dao 8, Hongqiao District, Tianjin 300130, China; dengyuchen816@163.com (Y.D.); lipeng@hebut.edu.cn (P.L.); 15122485497m@sina.cn (T.W.)

Correspondence: wangyige@hebut.edu.cn (Y.W.); lihuanrong@hebut.edu.cn (H.L.); Tel.: 86-22-60203674 (Y.W.); 86-22-60203674(H.L.)

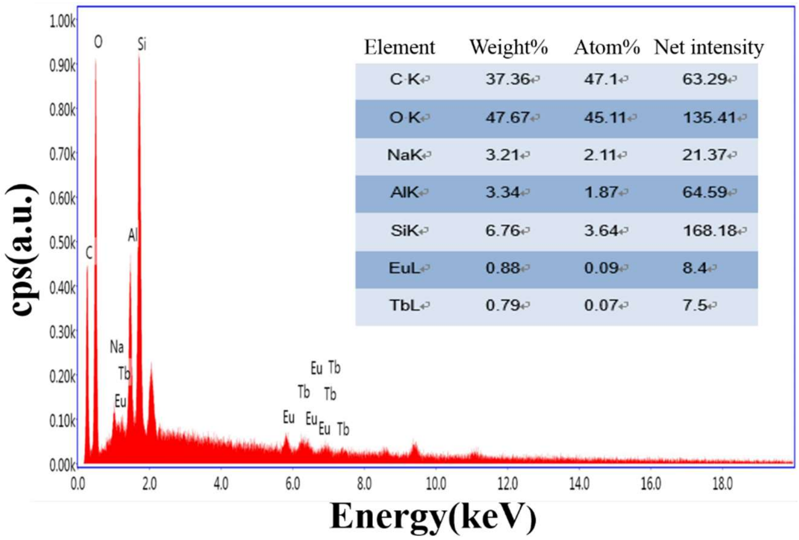

Figure S1. The EDX spectrum of $Eu_{0.5}Tb_{0.5}(HPBA_n)$@ZY.

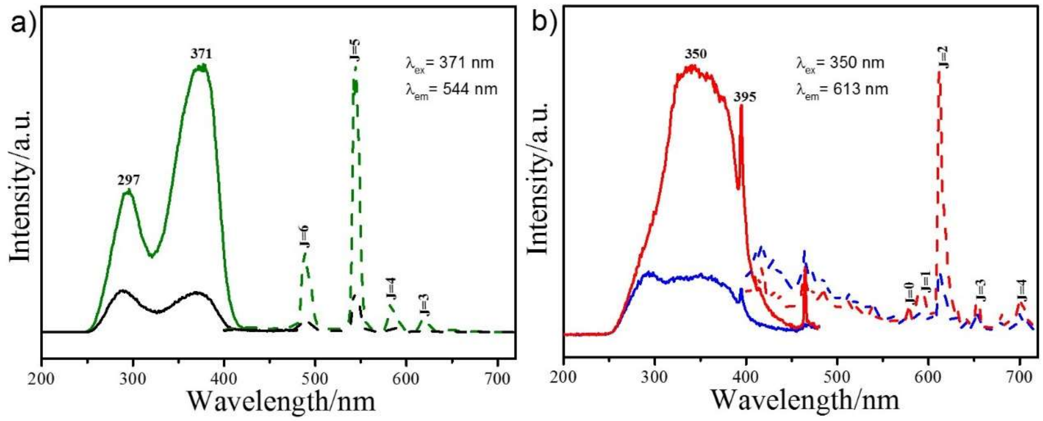

Figure S2. Excitation (green solid line) and emission spectra (green dot line) of $Tb(HPBA_n)$@ZY before treatment with NH_3 vapor; excitation (black solid line) and emission spectra (black dot line) of $Tb(HPBA_n)$@ZY after treatment with NH_3 vapor (a). Excitation (blue solid line) and emission spectra (blue dot line) of $Eu(HPBA_n)$@ZY before treatment with NH_3 vapor; excitation (red solid line) and emission spectra (red dot line) of

$Eu(HPBA_n)@ZY$ after treatment with NH_3 vapor (b).

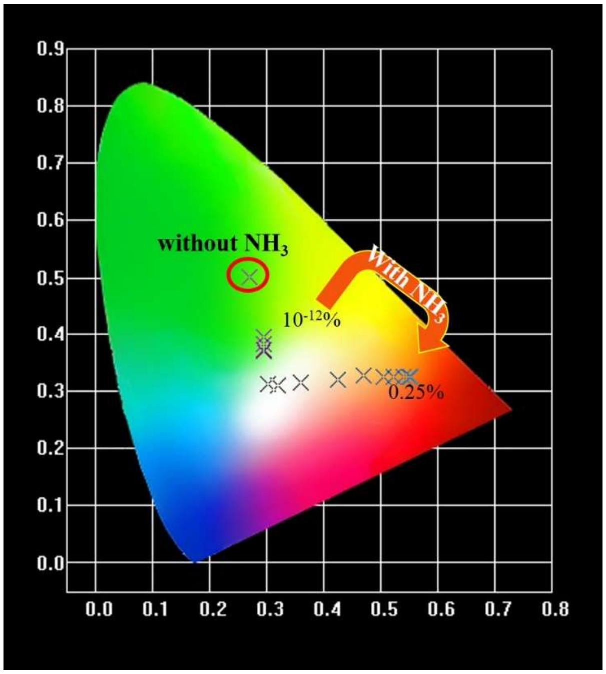

Figure S3. The corresponding CIE 1931 coordinates of $Eu_{0.5}Tb_{0.5}(HPBA_n)@ZY$ after treatment with different concentration of aqueous ammonia.

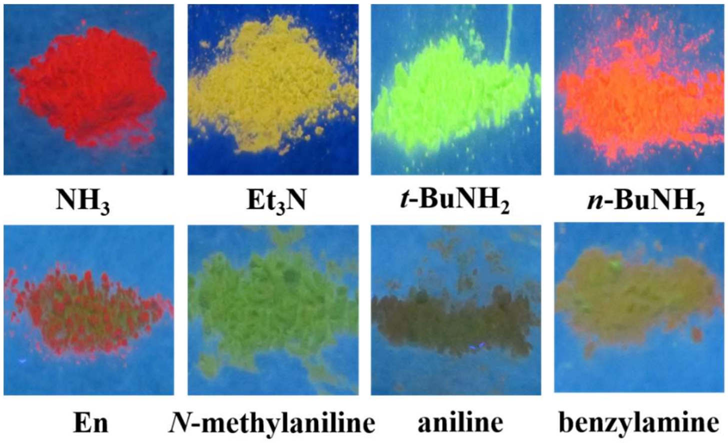

Figure S4. Digital photographs of $Eu_{0.5}Tb_{0.5}(HPBA_n)@ZY$ upon contact with amines for 10 min under near UV irradiation at 365 nm.

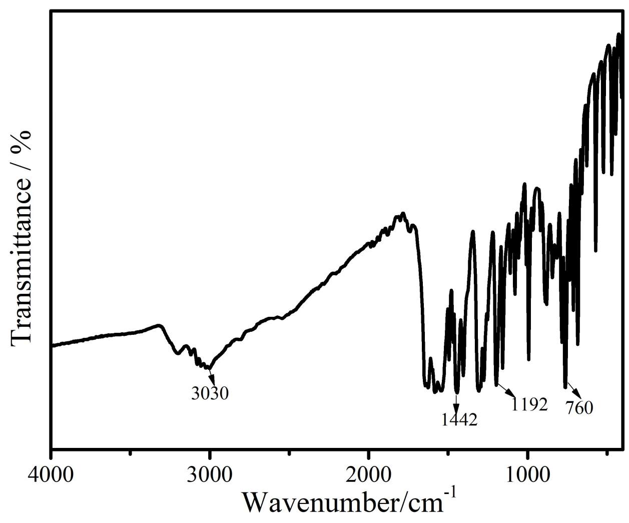

Figure S5. FTIR spectrum of HPBA.
